# Impact of Prehospital Ultrasound Training on Simulated Paramedic Clinical Decision-Making

**DOI:** 10.5811/westjem.18439

**Published:** 2024-06-28

**Authors:** Andrea Roche, Evan Watkins, Andrew Pettit, Jacob Slagle, Isain Zapata, Andrew Seefeld, Nena Lundgreen Mason

**Affiliations:** * Dartmouth College, Geisel School of Medicine, Department of Medical Education, Hanover, New Hampshire; † Speare Memorial Hospital, Department of Emergency Medicine, Plymouth, New Hampshire; ‡ New England College of Osteopathic Medicine, Biddeford, Maine; § Rocky Vista University, College of Osteopathic Medicine, Englewood, Colorado

## Abstract

**Introduction:**

When used appropriately, focused limited-scope ultrasound exams could potentially provide paramedics with accurate and actionable diagnostic information to guide prehospital decision-making. In this study we aimed to investigate the impact of a 13-hour prehospital ultrasound training course on the simulated clinical decision-making of paramedics as well as their ultrasound skills, knowledge, and self-confidence.

**Methods:**

We evaluated the ultrasound competence of 31 participants using post-course written and practical assessments. Written clinical decision scenarios were administered pre- and post-training. Post-training scenarios included an uninterpreted ultrasound clip to aid decision-making. Scenarios included extended focused assessment with sonography in trauma, pulmonary exam, and focused echocardiography combined with carotid pulse check exams. Correct answers to scenarios were defined as those selected by a veteran emergency physician. Participants also indicated their confidence in each of their decisions using a Likert scale.

**Results:**

Training yielded a statistically significant increase in both mean scenario score (35.5% absolute increase) and mean participant self-confidence (15.8% relative increase), across all exam/decision types assessed (*P* ≤ 0.001). The focused pulmonary exam yielded the largest increase in both mean score improvement (59.7% absolute increase) and paramedic confidence in their decisions (28.6% increase).

**Conclusion:**

Trained paramedics can perform focused ultrasound exams and accurately interpret and apply actionable exam findings in the context of written scenarios. Analysis through our model characterized the theoretical clinical yield of each prehospital ultrasound exam and demonstrated how each exam may provide improved decision accuracy in several specific simulated clinical contexts. These results provide support for growing evidence that focused limited-scope ultrasound may be an effective prehospital diagnostic tool in the hands of trained paramedics.

Population Health Research Capsule What do we already know about this issue?

*Appropriate prehospital treatment improves patient outcomes and reduces ED crowding.*

What was the research question?

*How does focused prehospital ultrasound training and knowledge affect paramedic clinical decisions in a simulated environment?*

What was the major finding of the study?

*There was a significant increase (p ≤ 0.001) in both mean scenario score (absolute 35.5%, from 55.1% to 90.6% correct)and mean self-confidence (6.0% absolute from 38 to 44%, 15.8% relative) across all exam/decision typesassessed. *

How does this improve population health?

*Focused, limited-scope ultrasound exams could provide paramedics with accurate and actionable diagnostic information to guide prehospital decision-making.*

## INTRODUCTION

Paramedics make critical prehospital treatment and transport decisions that often greatly impact patient outcomes. Appropriate prehospital treatment and receiving facility choices, as well as effective pre-arrival alerts, improve patient outcomes, decrease treatment cost, and reduce emergency department (ED) crowding.^1^ ^–^ ^3^ The prehospital environment is inherently complex and dynamic and can be resource limited, creating significant barriers to obtaining accurate diagnostic information needed to make appropriate decisions.^4^ ^–^ ^6^ While studies indicate that prehospital lung auscultation is 54% accurate^7^ and palpated carotid artery pulse check is 55% accurate,^8^ prehospital predictions of hospital care and clinical course are generally inaccurate.^9^ ^,^ ^10^ Diagnostic limitations render decision-making in the field difficult and can negatively impact patient outcomes. (For example, 22% of patients treated with prehospital needle decompression for tension pneumothorax were found to not have a pneumothorax after assessment in the ED.^11^)

Integration of advanced diagnostic tools in the prehospital setting has historically been successful in reducing decision barriers and improving outcomes. Use of electrocardiograms improved prehospital diagnostic positive predictive value for acute myocardial infarction from 33% to 93%.^12^ Point-of-care ultrasound (POCUS) is an advanced diagnostic tool that has immense potential to improve the accuracy of prehospital decision-making. Limited-scope POCUS is being implemented in emergency medical services (EMS) agencies across the United States and internationally. In 2014, 4.1% of responding EMS medical directors reported use of POCUS by their agencies, and 21.7% were considering future implementation.^13^ Common prehospital ultrasound (PHUS) exams include the extended focused assessment with sonography in trauma (eFAST), pulmonary exam, and focused echocardiography during cardiac arrest. When used appropriately, these exams can provide paramedics with accurate and actionable diagnostic information to guide prehospital decisions.^14^

For limited-scope PHUS to be safely implemented, we need a comprehensive understanding of three critical elements: paramedic performance; paramedic interpretation of POCUS exams; and the appropriate application of those exam findings to support prehospital decisions. Previous studies have demonstrated successful exam acquisition and interpretation by paramedics, such as eFAST, with 100% interpretation accuracy in a mean 2.6-minute exam time,^15^ lung ultrasound with a sensitivity of 80% and specificity of 72% for detecting pulmonary edema,^16^ and 88% accurate image interpretation of echocardiography during cardiac arrest.^17^ To our knowledge, no study has examined how POCUS training and education impact a paramedic’s ability to appropriately integrate PHUS exam findings into prehospital care in a simulated environment. Training paramedics to appropriately apply findings is essential to safe implementation of these skills in the field.^14^ A thorough understanding of how PHUS findings impact decisions about prehospital treatment, receiving facility choice, and pre-arrival alert is needed prior to safe and effective implementation of POCUS in the prehospital setting.

We examined how a hands-on PHUS training program impacted accuracy of simulated paramedic decision-making regarding prehospital treatment, receiving facility choice, and pre-arrival alerts using written, clinical decision scenarios administered pre- and post-training. The scenarios included eFAST, pulmonary exam, and focused echocardiography combined with carotid pulse check exams. In this study we also examined the impact of PHUS training and imaging on paramedic self-confidence in their simulated clinical decisions. To add context regarding the effectiveness of the education provided by the PHUS training administered, we also report the performance of the participants on course assessments including a written knowledge exam and scenario-based practical exams.

## METHODS

### Study Design and Equipment

This was a prospective observational cohort study designed to evaluate the effectiveness of a small-scale, mixed-modality (containing asynchronous digital independent prework, hands-on scanning practice, and clinical application scenarios) training program. The 13-hour course covered limited-scope POCUS exams that were focused specifically on aspects and applications of the exams with relevance to prehospital care. Exams included were the eFAST, focused pulmonary exam, and focused echocardiography combined with focused vascular exam for Doppler and visual carotid artery pulsatility in cardiac arrest. This study was approved and given an exempt determination by the Institutional Review Board Committee for the Protection of Human Subjects at Dartmouth College (#00032581). Ultrasound machines used in the course sessions included Clarius HD portable ultrasound units (Clarius Mobile Health Corp, Vancouver, BC, Canada), and Butterfly iQ+ (Butterfly Network Inc, Burlington, MA).

### Setting and Participants

New Hampshire has a population of 1.39 million with >1,100 licensed paramedics working in the state.^18^ ^,^ ^19^ New Hampshire poses a unique challenge to EMS systems, given its rural geography with often extended travel times to tertiary care centers. Participants in this study were primarily full-time paramedics licensed in New Hampshire. Participants were recruited for two in-person PHUS courses delivered on two different days through advertisement via the New Hampshite Division of Fire Standards and Training and Emergency Medical Services mailing list and website. Participants were given an overview of the study, provided an option to opt-out, and signed written consent forms. Thirty-one participants were enrolled. We collected participant experience-level, demographic, and employment data in a post-training survey.

### Course Procedure

The New Hampshire Fire Academy & EMS hosted the course at their training facility in. The was not involved in planning or conducting this study and did not financially support any members of the study team to conduct this research project. The course and its materials (Appendix 2) were assembled, created, overseen, and delivered by a board-certified emergency physician and ultrasound expert who also serves as an EMS medical director in conjunction with paramedics. This physician also selected and trained a small team of instructors with expertise in ultrasound imaging, paramedicine, and ultrasound education to assist him in delivering the course. Course objectives included learning appropriate indications for PHUS, ultrasound transducer manipulation, comfort with the user interface, ability to interpret results from selected POCUS exams, and ability to apply findings to clinical decision scenarios relevant to prehospital care. Specific details regarding course structure, content, and student/instructor ratios can be viewed in Appendix 2.

Prior to the in-person component, participants were provided with three hours of asynchronous education consisting of an introduction to relevant physics, use of ultrasound units, integration of ultrasound into workflow, selected POCUS exams, and cases. The in-person component of the course consisted of short didactic lectures, ample time for hands-on practice using live models, group discussion of cases, and standardized scenarios. Lectures reviewed selected POCUS exams and examples of pathology relevant to prehospital care. An instructor provided guidance and real-time feedback to participants in small groups. Content was reinforced and discussed in a large-group setting using integration questions and polling software.

### Measurements

#### Course Assessments

Participants ended the in-person course by taking a written knowledge test and completing six scenario-based exams covering cardiac arrest, respiratory distress, and trauma in EMS cases as part of a practical exam (Appendix 2). For the practical, participants performed selected POCUS exams on live models in small groups and received predetermined results via uninterpreted ultrasound video clips after successfully completing the exam. Scenario stations were operated by course instructors, and participants received “pass” or “fail” grades based on pre-established criteria for performance, image interpretation, and treatment/transport decisions. Case-based scenarios were completed as a team to simulate the prehospital environment, although each participant had to individually acquire a cardiac, lung, and eFAST exam on a model with normal findings and subsequently interpret a unique ultrasound image with pathology displayed on a computer screen and then apply findings to their treatment and transport plan without input from other team members. The post-course written exam consisted of 24 multiple-choice questions mapped to course objectives (topics covered in written exam questions can be viewed in Appendix 2). A score of ≥80% was set as the passing threshold for the written portion of the post-course exam.

#### Clinical Decision Scenarios

Prior to receiving educational content, participants were given written clinical decision scenarios (Table 1 and Appendix 1). This instrument was designed to measure the impact of PHUS training and availability of uninterrupted ultrasound images on paramedic clinical decisions. The instrument consisted of 10 vignettes that were intentionally ambiguous, reflecting the reality of prehospital emergency care. Scenarios provided an extensive description of the scene, patient assessment and history of present illness/injury, and were edited by multiple investigators.

**Table 1.** Clinical decision topics and the type of prehospital ultrasound exam included within each written, clinical decision scenario listed by scenario number. Each scenario number correlates with the number listed in Appendix 1, which shows the complete text of each scenario.

Scenario number	PHUS exam assessed	Scenario type	Clinical decision questions
Prehospital treatment scenarios			
2	eFAST	Trauma	Needle decompression vs no needle decompression in possible pneumothorax
4	eFAST	Trauma	TXA infusion vs no TXA with an unclear bleeding source
3	Focused pulmonary	Respiratory	Treatment of CHF vs COPD
5	Focused pulmonary	Respiratory	Treatment of CHF vs COPD
7	Echo + carotid pulse	Cardiac arrest	Continuation vs termination of resuscitation
8	Echo + carotid pulse	Cardiac arrest	Continuation vs termination of resuscitation
Transport and pre-arrival alert scenarios			
1	eFAST	Trauma	Trauma center vs closest ED, ground vs air transport, pre-arrival trauma alert vs no alert
6	eFAST	Trauma	Trauma center vs closest ED, ground vs air transport, pre-arrival trauma alert vs no alert
9	eFAST	Trauma	Trauma center vs closest ED, ground vs air transport, pre-arrival trauma alert vs no alert
10	eFAST	Trauma	Trauma center vs closest ED, ground vs air transport, pre-arrival trauma alert vs no alert

*PHUS,* prehospital ultrasound; *eFAST*, extended focused assessment with sonography in trauma; *TXA*, tranexamic acid; *CND*, chest needle decompression; *CHF*, congestive heart failure; *COPD*, chronic obstructive pulmonary disease; *ED*, emergency department; *POCUS*, point of care ultrasound.

Editing investigators included an emergency physician and ultrasound expert who also serves as an EMS medical director; a paramedic with over 20 years of experience in prehospital EMS education; and several paramedics, advanced emergency medical technicians (EMT), and EMT-Bs with field experience. Each scenario included one or more questions about prehospital treatment, receiving facility choice and transport modality, and/or pre-arrival alert. Scenario-based decisions were designed to specifically map to an associated element of the New Hampshire EMS protocols.

Participants also indicated their confidence in their decisions using a 1–5 Likert scale. Post-training, participants were given the same instrument, with the addition of an uninterpreted ultrasound clip that could be feasibly acquired in the field as a decision aid in each scenario. Correct answers were defined as those selected by a veteran emergency physician with expertise in both POCUS and EMS, who reviewed each clinical decision scenario with and without the associated ultrasound clip to establish the correct decisions for each scenario regardless of availability of ultrasound images. For scenarios where transport to a trauma center was indicated as correct, ground and air ambulance transport to a trauma center were both considered as correct answers to reduce scenario-based error around air ambulance availability, weather conditions, and specific location, etc. (Appendix 1).

### Data Analysis

We calculated descriptive statistics for categorical variables as frequencies with their respective percentages. Pre- vs post-score improvement was expressed in percentages; however, all assessments for association performed on the pre vs post were performed using paired *t*-tests on the actual scores. We evaluated normality assumptions in a preliminary evaluation of the distribution of the score and confidence variables using graphical methods (boxplots and histograms); no major concerns with meeting the normality assumptions were observed. We used non-parametric Wilcoxon rank-sum tests for pre vs post confidence-assessments scores. This was to accommodate for Likert scales used. Confidence assessments are expressed as sums and are displayed with their respective interquartile ranges. Associations to previous experiences with ultrasound were assessed through linear models where previous experience was coded as a categorical variable. Preliminary power analysis was not performed because participants were recruited via convenience sampling. We performed all analyses using SAS/STAT v.9.4 (SAS Institute Inc, Cary, NC). Significant differences were declared at *P* ≤ 0.05, although exact *P*-values are presented.

## RESULTS

### Participant Demographics

Of the 31 participants in this study, 30 completed the demographics survey (Table 2). A majority of the participants came from fire departments or private EMS agencies serving rural areas or small towns. A majority of paramedics were highly experienced with >63% having more than 16 years in EMS. Nine of the 30 paramedics reported having prior ultrasound experience in some capacity, three of whom described their training as specific to PHUS. Prior ultrasound experience consisted of vascular access training for all but two participants who reported more in-depth prior training. During analysis no significant associations were found between participants’ prior ultrasound or EMS experience and performance on course assessment or written clinical decision scenarios.

**Table 2.** Participant demographics and employment context survey. Participant-reported data regarding their prior experience working in emergency medical services and with ultrasound imaging.

Category	Frequency (%) N = 30^*^
EMS agency type	
Fire department	17 (56.7)
Private organization	4 (13.3)
Hospital	8 (26.7)
Air medical	1 (3.3)
Primary service provided	
911 with or w/o transport capability	21 (70.0)
Interfacility transport	1 (3.3)
Equal mix of 911 and interfacility transport	6 (20.0)
Clinical services	1 (3.3)
Mobile integrated healthcare and community paramedicine	1 (3.3)
Years of EMS experience	
>21 years	16 (53.3)
16–20 years	3 (10.0)
11–15 years	5 (16.7)
8–10 years	3 (10.0)
5–7 years	3 (10.0)
Size of community served	
Rural (<2,500)	5 (16.7)
Small town (2,500–24,999)	19 (63.3)
Medium-size town (25,000–74,999)	6 (20.0)
Prior ultrasound experience	
Received prior training–any capacity	6 (20.0)
Received prior training-specifically for EMS use	3 (10.0)
No prior training	21 (70.0)
Prior ultrasound use on the job	
Prior use–any capacity	4 (13.3)
Prior use–specifically in EMS job	2 (6.7)
No prior use	24 (80.0)

* 30 of the 31 study participants are represented, (One participant did not complete the employment context survey.)

*EMS*, emergency medical services.

### Course Assessments

Of the 31 (87.1%) participants, 27 obtained a passing grade on the written post-course exam. The cohort average score was 92.2%, with a range of 62.5–100%. The scenario-based practical exam had a 100% pass rate.

### Clinical Decision Scenarios

Data depicting the comparison of the pre- and post-course written, clinical decision scenarios are shown in the Figure. The addition of PHUS imaging yielded a statistically significant increase in both mean score and mean participant self-confidence across all PHUS exam types and decision types assessed by this instrument (*P* ≤ 0.001). The pulmonary exam yielded the largest increase in both mean score improvement (59.7%) and paramedic confidence in their decisions (28.6%). The smallest increases in improvement were observed in the echo/carotid pulse exam categories at 29% and 12.5%, respectively. When comparing changes in prehospital treatment, transport, and receiving facility decisions, the largest increase in mean score and confidence was seen in prehospital treatment choices. Of the 14 scenario questions answered by all 31 participants (434 unique answers), 168 answers (38.7%) were changed from incorrect to correct with the aid of ultrasound images.

**Figure.**
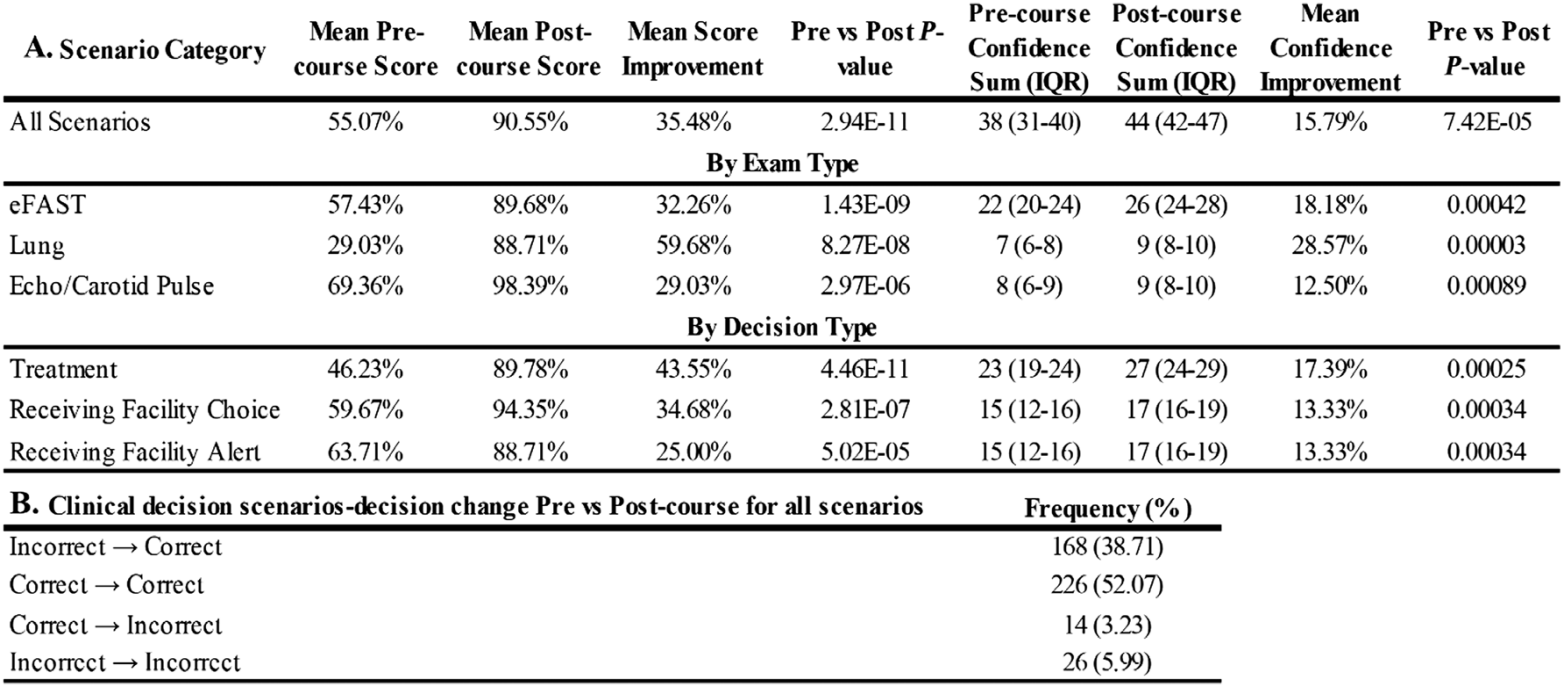
Changes in paramedics’ simulated clinical decision-making and self-confidence following training. Panel A depicts the mean improvement in both mean score and participant self-confidence associated with each written clinical decision scenario (Table 1 and Appendix 1). Scores are sorted by specific prehospital ultrasound (PHUS) exam type and clinical decision type. Panel B reports how aggregate participant responses to all 10 scenarios changed with regard to correctness when they had access to uninterpreted PHUS images as a decision aid. All changes are highly significant at *P* ≤ 0.001. *IQR*, interquartile range; *eFast*, extended focused assessment with sonography in trauma.

### Previous Participant Ultrasound Experience

Participant previous experiences with ultrasound are presented in Table 2. Only 9 of 30 participants reported having any type of previous experience using or being trained with ultrasound imaging and only two individuals reported having used ultrasound on the job before participation in this study. We used linear models to look for associations between any type of prior experience with ultrasound that a participant had, and their performance on study assessments; however, no associations were detected for any of the previous ultrasound experience-types reported.

## DISCUSSION

### Course Assessments

The post-course written test evaluated participant mastery of the course objectives; 87.1% of participants passed the test with an average score of 92.2%, demonstrating that the course educated paramedics in basic theory, knowledge, and interpretation of the three, goal-directed, limited window PHUS exams. This result supports a growing body of evidence that 1–2 days of instruction across a variety of instructional modalities and exam types is largely adequate for paramedics to achieve competency with limited scope PHUS exams.^17^ ^,^ ^20^ ^–^ ^23^

The practical test evaluated participant psychomotor skills, as well as the ability to integrate PHUS into EMS workflow, accurately interpret exams, and appropriately apply findings in real time. One hundred percent of participants passed the practical, demonstrating that the course successfully trained paramedics to acquire, interpret, and integrate PHUS into practical EMS scenarios. This supports previous evidence that paramedics can acquire and interpret PHUS images in the field.^15^ ^–^ ^17^ ^,^ ^24^

### Clinical Decision Scenarios

The written clinical decision scenarios used in this study present a novel way to evaluate the impact of PHUS on paramedic decision-making in a risk-free manner. The pre-course scenarios did not include ultrasound images and required the paramedic to make difficult and potentially ambiguous decisions based on history, physical exam, and conventional diagnostics alone, accurately mimicking the difficulty of real decisions made on shift. Many studies have shown the inaccuracy of conventional prehospital diagnostics and highlight the difficulty in predicting patient condition and disposition based on prehospital history and physical exam alone.^1^ ^,^ ^5^ ^–^ ^10^ Access to uninterpreted, raw ultrasound clips that the participants were trained to acquire and could feasibly obtain in the field yielded a statistically significant increase in correct decision making in every category evaluated, including treatment, transport, and pre-arrival alert. Mean scores also increased significantly in all types of PHUS exams evaluated (Figure). Despite potential sources of error described above, these results provide a compelling theoretical framework to analyze how PHUS may impact paramedic decisions.

#### Focused Pulmonary Exam

Access to focused pulmonary ultrasound across respiratory distress scenarios (3 and 5) improved decision accuracy in prehospital treatment by 59.7% and confidence by 28.6% (Figure). Scenarios 3 and 5 required participants to determine whether to follow the congestive heart failure (CHF) or chronic obstructive pulmonary disease (COPD) treatment protocols in a patient in respiratory distress of unclear etiology suspected to have CHF vs COPD. Access to uninterpreted PHUS significantly improved these decisions. This result supports existing data that paramedics can accurately interpret lung ultrasound in the setting of pulmonary edema.^16^ It also indicates that in the unclear circumstance of a respiratory distress patient where CHF vs COPD is suspected, focused pulmonary ultrasound may provide improved accuracy of paramedic working diagnosis, increased confidence, and accuracy of prehospital treatment in this specific clinical setting. The ability of paramedics to appropriately apply findings to support simulated decisions indicates that the improved diagnostic accuracy of pulmonary ultrasound in undifferentiated dyspnea demonstrated outside the US may also be applicable in the prehospital context within the US.^25^

#### Extended Focused Assessment with Sonography in Trauma

Access to eFAST images in trauma (scenarios 1, 2, 4, 6, 9, 10) improved overall decision accuracy by 32.3% and confidence by 18.2% (Figure). This result theoretically supports previous studies that paramedics can accurately interpret an eFAST exam.^15^ ^,^ ^24^ It also indicates that eFAST may improve paramedic accuracy in determining the appropriate transport method and receiving facility type in complex trauma patients. Similarly, eFAST may improve the accuracy of needle decompression in this specific clinical setting, which may help to reduce the demonstrated incidence of unnecessary prehospital needle decompression, as well as improve the appropriate use of tranexamic acid infusion in unclear circumstances such as when an intra-abdominal bleeding source is not obvious on physical exam.^11^

#### Focused Echocardiography and Point-of-care Ultrasound (Carotid) Pulse Check

Access to focused echocardiography and POCUS (carotid) pulse check (scenarios 7 and 8) significantly improved mean decision accuracy by 29%, and confidence by 12.5%. Scenarios represented a pulseless electrical activity (PEA) or asystole cardiac arrest case where termination parameters were met by a small margin. Pseudo-PEA was a feasible possibility in scenario 7. Access to PHUS improved the accuracy of appropriate termination of resuscitation in the asystole scenario (8) as well as appropriate continuation of resuscitation in the PEA scenario, which was actually pseudo-PEA as identified by ultrasound exam.

This result demonstrates that paramedics can accurately interpret these exams in the setting of simulated cardiac arrest, supporting existing data on prehospital echocardiography interpretation.^17^ ^,^ ^24^ Additionally, focused echocardiography and carotid pulse check in cardiac arrest may improve paramedic accuracy in determining whether and when termination of resuscitation is appropriate. These results also provide a novel theoretical representation that PHUS may be effective in identifying and acting on prehospital pseudo-PEA cardiac arrest. Lastly, the result demonstrates that access to PHUS in cardiac arrest may improve paramedic confidence in resuscitation decisions, which can often be difficult and stressful.^17^

Although significant, these changes in mean score and confidence are the smallest in magnitude that we observed. This may have been due to increased complexity and difficulty in image acquisition and interpretation of cardiac images as compared to other PHUS exams. Although results demonstrate that paramedics can perform and then interpret and apply simulated findings from a focused vascular exam for presence or absence of carotid pulse as an adjunct to cardiac arrest echocardiography, this is in the context of a purely simulated theoretical environment and does not provide any insight into the clinical validity, utility, or ideal method of carotid pulse check, which remains an area of active study.^26^ ^,^ ^27^

#### Integration of Prehospital Ultrasound

As PHUS is implemented, its safety and efficacy will depend on a thorough understanding of how paramedics apply exam findings to prehospital treatment decisions. The current body of evidence has not yet established the clinical yield, benefits, and risks regarding each PHUS exam type. Understanding which exams result in significantly improved accuracy of decisions that lead to an actionable change in prehospital management is a crucial next step. Judicious and precise integration of exams that are proven to have good prehospital yield may have the potential to improve patient care through improved diagnostic accuracy. Conversely, inaccurate application of PHUS test characteristics, incongruence with the overall clinical picture, or imaging resulting in unactionable information may result in poorly applied exam findings with risks such as overtriage, extended assessment time, or deviation from existing standards of care. Analysis of PHUS, like the theoretical model in this study, may help to inform paramedic education and protocols needed to ensure that PHUS findings are applied in a manner that improves decisions and minimizes these potential harms.

## LIMITATIONS and FUTURE DIRECTIONS

This study used a relatively small sample size of 31 participants. It examined participant data across two repeated PHUS courses with identical curricula, the same course director, and similar instructors. There is potential variability between the two course sessions due to uncontrollable factors such as course dynamics or participant interaction or varying prior experience with ultrasound imaging and EMS. The three hours of course assigned prework were completed on the honor system, and the study team could not verify completion of that prework. This course was publicly advertised to paramedics; thus, self-selection bias could have influenced those who participated by attracting paramedics with more experience and advanced training. The clinical decision scenarios were a theoretical framework to simulate real-world prehospital care. As with any such instrument, there are potential sources of error such as variable simulation fidelity and potential misinterpretation of depicted scenes.

The repeated use of the same written clinical decision scenarios before and after intervention without a control group may have introduced potential confounding. Because the post-training scenarios contained additional information in the form of ultrasound imaging, it is difficult to determine whether changes in paramedic accuracy were influenced by repeated assessment, the additional imaging, the training itself, or a combination of these factors. Additionally, some of the clinical decision scenarios are written in a manner that may have flagged the uninterpreted ultrasound image as abnormal. However, the participants were still required to identify the type of positive findings in the image attached to each scenario, and correctly apply that information within the clinical context of each vignette.

Lastly, none of the models possessed pathology; therefore, study participants were not able to scan pathology themselves during the course. Rather, relevant pathological images were covered thoroughly during the didactic sessions of the course. In terms of previous ultrasound experience affecting participant scores, the cohort was limited in its capacity to detect such associations. This could be because of low power due to a small portion of the participant having previous experience with ultrasound in general or because those effects are small in the large context of this prehospital application.

Further study in the field is necessary to expose paramedics to the typical distractions and suboptimal imaging conditions they will experience in the field to validate these theoretical, scenario-based findings, and to continue classifying the clinical yield and decision support of PHUS, to guide the development of PHUS protocols and best practices. Further study is also needed to characterize PHUS knowledge and skill retention over time.

## CONCLUSION

This study showed that with a 13-hour mixed modality training program, paramedics can competently perform focused eFAST, pulmonary, and cardiac arrest ultrasound exams during course assessments. They can also accurately interpret exam findings and apply these actionable findings within a scenario context resulting in a theoretical significant increase in decision accuracy and potential improvement in prehospital care. Decision analysis through our clinical decision scenarios model characterized the theoretical clinical yield of each focused PHUS exam and demonstrated how each exam may provide improved decision accuracy in several specific clinical contexts. These results provide support for growing evidence that focused eFAST, pulmonary, and cardiac arrest ultrasound may be safe and effective prehospital diagnostic tools in the hands of trained paramedics.

## Supplementary Information




