# Supplementary material for: Impact of Prehospital Ultrasound Training on Simulated Paramedic Clinical Decision-Making
Source: West J Emerg Med. 2024 Jun 28;25(5):784–92. doi: 10.5811/westjem.18439 (PMC11418876; doi:10.5811/westjem.18439)
Supplement: Supplementary file 1 [file wjem-25-784-s001.docx]

**Scenario 1**

You are on scene with a 45 y/o Male patient who was the single restrained occupant of a MVA involving a passenger vehicle vs tree at 45mph that occurred 20 minutes ago. The vehicle did not roll and has moderate front end damage with airbag deployment and no passenger compartment intrusion. The patient is alert and oriented with a GCS of 15, denies loss of consciousness, and provides a history of swerving off the road and impacting the steering wheel during the MVA. His only complaint is 5/10 dull right-sided, non-radiating abdominal pain that has been consistent since the MVA. Physical exam reveals redness and some contusion to the RUQ and right flank, no other signs of trauma are present. Abdominal exam reveals tenderness to the RUQ and RLQ, voluntary right-sided guarding due pain, and no abdominal rigidity. Breath sounds are clear and equal bilaterally, pupils are equal and reactive to light, skin appears normal, and the patient moves all extremities normally. The patient denies any medical history, current medications, or allergies. Vitals are: BP 136/88, HR 114, RR 20, SpO2 95%

*Question 1:* Based on the available information, the most appropriate destination/disposition for this patient is:

1. Ground ambulance transport to Small Local ED (no NH trauma system designation, 20 minute drive)
2. **Ground ambulance transport to Regional Trauma Center (NH Level 2 trauma center, 60 minute drive)**
3. **Air ambulance transport to Regional Trauma Center**
4. Patient self-transport to destination of choice

*Question 2:* Appropriate receiving facility pre-arrival notification includes:

1. **Trauma alert**
2. No pre-arrival notification beyond standard call, the patient does not meet alert criteria

*Confidence Scale:* My confidence in this decision using the available information is:

1. Not confident in the decision, I need more information than provided to make a confident decision

2.

3.

4.

5. Confident, the information provided was all I need to make a confident decision

*Post-Course Ultrasound Image:* Video clip of RUQ eFAST window with positive findings of free fluid in Morrison’s pouch. Text stating “negative findings in all other windows”

**Scenario 2**

You are on scene with a 32 y/o helmeted Male who was involved in a rollover ATV accident on a dirt road at 30mph that occurred 15 minutes ago. The patient is found 20ft down an embankment near the ATV which has moderate damage. The patient is alert and oriented with a GCS of 15, denies loss of consciousness, and provides a history of losing control of the ATV, rolling with the ATV off the road, and being crushed by the ATV on his left side during the rollover. The patient is yelling in pain, providing limited information and complaining “my entire left side hurts and it hurts to breathe” pointing from his anterior left chest to his left pelvis. The patient denies any medical history, current medications, or allergies. On physical exam the patient is alert but appears agitated and anxious, and is breathing with rapid, shallow breaths. There is obvious contusion to the left anterior/lateral chest, left flank, and left abdomen. The left rib cage is tender with crepitus and what appears to be deformity to inferior left lateral ribs though it is difficult to see through the chest wall adipose tissue. The trachea is midline and JVD is not present. The abdomen is diffusely rigid and tender. The pelvis is intact and non-tender. No other signs of trauma are present. Pupils are equal and reactive. Skin is diaphoretic and appears pale. Breath sounds are difficult to auscultate due to the patient's rapid/shallow breathing and scene noise, and sound intact but faint bilaterally. Vitals are: BP: 98/60, HR: 146, RR: 28, SpO2 88%, EtCO2 42.

Question 1: Appropriate treatment for this patient includes:

A. Left-sided needle decompression for suspected tension pneumothorax

**B. Does not include needle decompression, tension pneumothorax is not suspected**

*Confidence Scale:* My confidence in this decision using the available information is:

1. Not confident in the decision, I need more information than provided to make a confident decision

2.

3.

4.

5. Confident, the information provided was all I need to make a confident decision

*Post-Course Ultrasound Image:* Video clip of RUQ eFAST window with positive findings of free fluid in Morrison’s pouch Text stating “negative findings in all other windows”

Video clip of bilateral anterior lung fields showing bilateral lung sliding present

**Scenario 3**

You are called to residence for a 74 y/o Female with difficulty breathing. The patient is alert and oriented and states that she has had worsening shortness of breath over the last 8 hours that began gradually after waking up this morning. She called EMS because she is now too short of breath to walk around the house which she can do at baseline with ease. She denies chest pain, dizziness or other symptoms today and denies any recent sickness, cough/sputum, or changes in health. She reports a history of COPD with an albuterol inhaler that she tried once earlier today and says “I’m not really sure if it helped or not.” She also reports a history of CHF, sleeps in an armchair and has baseline lower extremity edema. She reports a previous history of 1-2 similar episodes of shortness of breath in the last 3 years and states “I've had flare ups with my lungs and my heart but I’m not really sure.” Her medications include metoprolol, lisinopril, furosemide, albuterol, and a long-acting inhaler. On physical exam the patient is alert and conversing with difficulty through obvious shortness of breath. Lung exam reveals wheezing in bilateral lower lung fields. Equal peripheral edema bilaterally in lower extremities. Skin appears normal. Vitals are: BP 164/98, HR: 68, RR: 30, SPO2 84%, EtCO2 44, 12-lead EKG shows normal sinus rhythm.

*Question 1:* Appropriate treatment for this patient includes: (Likert Scale)

**A. Oxygen, IV access, consider CPAP, consider IV/SL nitroglycerin titrated to blood pressure for suspected CHF exacerbation**

B. Oxygen, IV access, nebulized albuterol/ipratropium, consider CPAP, consider steroids for suspected COPD exacerbation

C. Oxygen and IV access only

*Confidence Scale:* My confidence in this decision using the available information is:

1. Not confident in the decision, I need more information than provided to make a confident decision

2.

3.

4.

5. Confident, the information provided was all I need to make a confident decision

*Post-Course Ultrasound Image:* Video clips of left and right posterior lung fields, both with diffuse b-lines present

**Scenario 4**

You are called to a local ski area for a 63 year old helmeted female who lost control while skiing at an unknown speed and collided with a tree 20 minutes ago. She is laying on a cot in the ski patrol treatment area and obviously anxious and in pain. She is alert and oriented with a GCS of 15 and provides a history of skiing, losing control, and colliding with the tree, impacting her left side and left lower extremity. She denies loss of consciousness or preceding symptoms. She reports isolated 8/10 worsening left lower extremity pain at the top of her boot and denies any other symptoms. She reports a history of a deep vein thrombosis 2 months ago for which she takes apixaban (Eliquis), but otherwise reports being “very healthy” with no other PMHx, medications, or allergies. Physical exam reveals a closed bony deformity to the left tibia/fibula at the top of her ski boot with distal circulation and sensation intact and no other trauma to the left leg. Many small contusions to the left shoulder, arm, flank, and hip are noted. Tenderness is present to the general left arm and left flank though distracted by her leg pain. Her skin is diaphoretic and pale. Physical exam is otherwise unremarkable with no other signs of trauma. Pupils are equal and reactive, breath sounds clear, abdomen soft and nontender, pelvis is intact and non-tender. Vitals are: BP: 94/58, HR: 140, RR: 28, SpO2 93%.

*Question 1:* Appropriate treatment for this patient includes:

**A. A 1g tranexamic acid infusion, the patient meets TXA criteria per protocol**

B. Does not include a TXA infusion, the patient does not meet TXA criteria

*Confidence Scale:* My confidence in this decision using the available information is:

1. Not confident in the decision, I need more information than provided to make a confident decision

2.

3.

4.

5. Confident, the information provided was all I need to make a confident decision

*Post-Course Ultrasound Image:* Video clip of RUQ eFAST window with positive findings of free fluid in Morrison’s pouch Video clip of sagittal suprapubic eFAST window with positive findings of suprapubic free fluid Text stating “negative findings in all other windows with normal lung sliding bilaterally”

**Scenario 5**

You are called to a residence for a 70 year old male with shortness of breath. The patient is alert and oriented and states that he has been increasingly short of breath and fatigued for the last 2 days. He reports with these symptoms he has noticed increased lower extremity edema above baseline. He reports his shortness of breath progressively worsened today to dyspnea at rest in bed so he called EMS. He denies any other symptoms or changes in health. He reports a 5-year history of CHF for which he takes lisinopril, metoprolol, spironolactone, and furosemide. He also reports a 20-year history of COPD for which he takes a long-acting bronchodilator and steroid as well as an albuterol inhaler as needed that he reports “hasn’t really changed” his symptoms in the last few days. He denies other history, medications or allergies. On physical exam the patient is alert and conversing with apparent difficulty breathing. Lung exam reveals diffuse wheezing that is difficult to auscultate. Equal peripheral edema bilaterally in lower extremities. Skin appears normal. Vitals are: BP 170/100, HR: 70, RR: 30, SPO2 84%, EtCO2 46, Temperature 98.4 F, 12-lead EKG shows normal sinus rhythm.

*Question 1:* Appropriate treatment for this patient includes:

A. Oxygen, IV access, consider CPAP, consider IV/SL nitroglycerin titrated to blood pressure for suspected CHF exacerbation

**B. Oxygen, IV access, nebulized albuterol/ipratropium, consider CPAP, consider steroids for suspected COPD exacerbation**

C. Oxygen and IV access only

*Confidence Scale:* My confidence in this decision using the available information is:

1. Not confident in the decision, I need more information than provided to make a confident decision

2.

3.

4.

5. Confident, the information provided was all I need to make a confident decision

*Post-Course Ultrasound Image:* Video clips of left and right posterior lung fields, both with a-lines present and without any b-lines present

**Scenario 6**

You are called to a local park for an intoxicated 28 year old male that fell 20 minutes ago. The patient’s friends report the patient was walking on a concrete retaining wall when he stumbled off the wall and fell onto a park bench, though the story is unclear as they did not witness the fall and are also intoxicated. The patient is sitting in the grass next to a park bench at the bottom of a 6ft concrete wall, GCS 14. The patient is alert and oriented though obviously intoxicated and not very cooperative with conversation. He reports walking on the wall for fun “when I don’t know, I just tripped off, but I am fine” and does not provide any other history of the fall. He denies loss of consciousness, denies any current symptoms, denies any medical history, allergies, or medications, and reports drinking “at least 10 beers” in the last 4 hours. On physical exam the patient has superficial, non-bleeding abrasions to his right forehead and face and to his right abdomen and down his right thigh. Physical exam is otherwise unremarkable with no other signs of trauma. Pupils are equal and reactive, breath sounds clear, abdomen soft and tender over right-sided abrasions, pelvis is intact and non-tender, the patient moves all extremities well, skin is normal. Vitals are BP: 118/68, HR: 138, RR: 18, SpO2 94%, BGL: 122.

*Question 1:* Based on the available information, the most appropriate destination/disposition for this patient is:

A. Ground ambulance transport to Small Local ED (no NH trauma system designation, 20 minute drive)

**B. Ground ambulance transport to Regional Trauma Center (NH Level 2 trauma center, 60 minute drive)**

**C. Air ambulance transport to Regional Trauma Center**

D. Patient self-transport to destination of choice

*Question 2:* Appropriate receiving facility pre-arrival notification includes:

**A. Trauma alert**

B. No pre-arrival notification beyond standard call, the patient does not meet alert criteria

*Confidence Scale:* My confidence in this decision using the available information is:

1. Not confident in the decision, I need more information than provided to make a confident decision

2.

3.

4.

5. Confident, the information provided was all I need to make a confident decision

*Post-Course Ultrasound Image:* Video clip of RUQ eFAST window with positive findings of free fluid in Morrison’s pouch. Text stating “negative findings in all other windows with normal lung sliding bilaterally”

**Scenario 7**

You are called to the scene of a 78 y/o M whose wife has found him unresponsive in bed at 11 pm when she woke up to use the bathroom. She tried to rouse him and was unsuccessful, and noticed that he was not breathing. She called 911 immediately but could not perform chest compressions due to her age and disability. EMS arrived on scene after 12 minutes, spent 2 minutes getting access to the building and found the patient lying in bed, pulseless and apneic. The patient doesn’t have a DNR/DNI on file per his wife. EMS moved the patient from his bed to the floor and began CPR, noting an initial rhythm of coarse Vfib which was immediately defibrillated into asystole. You continue resuscitation and provide 1mg of 1:10,000 epinephrine after establishing IO access. Per his wife the patient has a significant cardiac history and has had 15 cardiac catheterizations. He had a below-the-knee amputation due to complications of diabetes. There are no signs of drug abuse or choking on scene and the patient’s wife says he was feeling well earlier in the day. The patient is atraumatic and there is no emesis in his airway, and you intubate without difficulty. His initial EtCO2 is 30 mm Hg. You confirm bilateral breath sounds and lack of epigastric sounds. After 3 pulse checks indicating asystole and pulselessness, you observe an organized narrow-complex rhythm on the monitor at a rate of 48 BPM. You cannot palpate a carotid or femoral pulse and cannot obtain a reading on your pulse oximeter. At the next pulse check you attempt to listen for heart tones with your stethoscope but don’t hear any. You continue to observe an EtCO2 of 32 mm Hg. At the 16-minute pulse check you have given 3 rounds of 1 mg of 1:10,000 epinephrine and continue to observe the same findings. You consider the use of sodium bicarbonate and/or calcium gluconate. 2 law enforcement officers and an additional EMS unit have arrived on scene.

*Question 1:* Based on the available information, your next action should be to:

A. Consult medical direction regarding termination of resuscitation on scene when criteria are met

**B. Consult medical direction regarding transport to the Local ED and continue resuscitation enroute**

*Confidence Scale:* My confidence in this decision using the available information is:

1. Not confident in the decision, I need more information than provided to make a confident decision

2.

3.

4.

5. Confident, the information provided was all I need to make a confident decision

*Post-Course Ultrasound Image:* Video clip of a short axis carotid pulse check with color flow doppler showing obvious pulsatile flow. Video clip of a subxiphoid echocardiogram window showing organized cardiac motion without pericardial effusion

**Scenario 8**

You are called to a rural private residence for a 58 y/o M who was found collapsed on the floor by his 16 y/o son. The son noted that his father wasn’t breathing and called 911, then performed chest compressions under the direction of the dispatcher while waiting for EMS to arrive. BLS EMS unit arrives on scene within 14 minutes and is backed up by a paramedic fly car shortly afterwards. EMS arrives at the home to find the patient’s son performing CPR on the patient, who has collapsed under a floating shelf in his kitchen and appears to have been on his laptop. There are no signs of trauma or drug use and the patient’s son denies DNR/DNI. The son is in distress and leaves the room. The patient is pulseless and apneic and there is emesis in the airway. EMS moves the patient to an open part of the kitchen to perform CPR and cuts off his shirt, revealing healed scars from an open sternotomy as well as a pacemaker implanted near his left shoulder. EMS initiates chest compressions and notes initial rhythm to be asystole with regular spikes from a dual-chamber pacemaker that is actively firing. EMS withholds defibrillation but obtains IO access and delivers 1 mg of 1:10,000 epinephrine. EMS clears the patient’s airway with suction and intubates using the SALAD method before securing an ET tube and ventilating the patient. Patency is confirmed with bilateral breath sounds, absent epigastric sounds and an EtCO2 of 15 mm Hg. The patient’s ex-wife arrives on scene and states that he has a history of IV drug abuse and endocarditis with valve replacement. You continue to perform CPR and give IV naloxone and 3 rounds of 1 mg of 1:10,000 epinephrine, and note an increase in EtCO2 to 24 mm Hg. During pulse checks, you continue to observe asystole with dual-chamber pacemaker firing and you cannot palpate carotid or femoral pulses. You cannot auscultate heart tones with a stethoscope or obtain a pleth with your pulse oximeter. It has been 16 minutes since EMS initiated resuscitation.

*Question 1:* Based on the available information, your next action should be to:

**A. Consult medical direction regarding termination of resuscitation on scene when criteria are met**

B. Consult medical direction regarding transport to the Local ED and continue resuscitation enroute

*Confidence Scale:* My confidence in this decision using the available information is:

1. Not confident in the decision, I need more information than provided to make a confident decision

2.

3.

4.

5. Confident, the information provided was all I need to make a confident decision

*Post-Course Ultrasound Image:* Video clip of a subxiphoid echocardiogram window showing lack of any organized cardiac motion and no pericardial effusion

**Scenario 9**

You are dispatched to a rural private residence for a 72 y/o Male who fell while working in his yard. It’s just above freezing and there is patchy snow and ice on the ground. Upon arrival you’re flagged down by the patient’s wife, who is standing in the driveway. She walks you around to the back of the house and explains that her husband fell from a stepladder while investigating an ice dam on the roof. She states that he’s been feeling ill for the past week with diarrhea and vomiting after a recent trip to Florida. You arrive to find the patient sitting on the back deck clutching his ribs. He states that he slipped on the 4’ step ladder and landed on the deck guardrail with his chest, then fell onto the deck. He has pain and “a twinge” in his neck, but denies back pain, loss of consciousness or head injury. He continues to feel nauseated and fatigued but states that he has been sick for several days. You note the patient to have an elevated respiratory rate and he describes feeling short of breath. He is fully alert and oriented (GCS 15) and has sensation and motion in all four extremities, and you do not observe any bleeding. You apply a C-collar and load the patient into the ambulance with spinal motion precautions, and on physical exam of the chest you see a large red mark over the nipple line where he landed on the guard rail. His wife hands you a plastic bag with pill vials and you note he takes apixaban (Eliquis), aspirin, diltiazem (Cardizem) and tamsulosin. You don’t feel any crepitus when palpating the chest wall but the patient reports 5/10 pain on deep inspiration. You hear clear lung sounds bilaterally and heart tones are audible near the apex. You do not observe tracheal deviation and the patient’s jugular veins are hidden underneath the C-collar at this point. Vitals are BP: 98/68, HR: 104, RR: 24, SpO2 97%, BGL: 91 mg/dL.

*Question 1:* Based on the available information, the most appropriate destination/disposition for this patient is:

A. Ground ambulance transport to Small Local ED (no NH trauma system designation, 20 minute drive)

**B. Ground ambulance transport to Regional Trauma Center (NH Level 2 trauma center, 60 minute drive)**

**C. Air ambulance transport to Regional Trauma Center**

D. Patient self-transport to destination of choice

*Question 2:* Appropriate receiving facility pre-arrival notification includes:

**A. Trauma alert**

B. No pre-arrival notification beyond standard call, the patient does not meet alert criteria

*Confidence Scale:* My confidence in this decision using the available information is:

1. Not confident in the decision, I need more information than provided to make a confident decision

2.

3.

4.

5. Confident, the information provided was all I need to make a confident decision

*Post-Course Ultrasound Image:* Video clip of a subxiphoid echocardiogram window showing large pericardial effusion with evidence of tamponade. Text stating “negative findings in all other eFAST windows with normal lung sliding bilaterally”

**Scenario 10**

You are called to a local ski area on a below freezing day for a 11 year old helmeted female who lost control at an unknown speed and collided with a trail sign 30 minutes ago. On arrival, the patient is lying in a cot, tearful and somnolent. The patroller tells you that one of her skis broke on impact and that she had a brief loss of consciousness prior to their arrival. She has retrograde amnesia and confusion since, but is able to answer some questions. The patient is complaining of 7/10, right lower leg pain and a mild headache. No vomiting or visual changes. Her father reports she has no notable medical history, medications, or allergies. Physical exam reveals a closed bony deformity on the lateral aspect of her right lower leg with swelling but no discoloration. Sensation and distal circulation are intact in all four extremities. Tenderness is present over the right lateral breast though the patient is distracted by her leg pain. Head is normocephalic and atraumatic. Pupils are equal and reactive. Breath sounds are clear in bilateral bases, but right apex is questionably reduced. The patient does not report difficulty breathing. Skin is cool, pale and clammy. Abdomen is soft and nontender, pelvis is intact and non-tender. No other signs of trauma. Vitals are: BP 108/72 HR: 115 RR:28 SpO2 95%.

*Question 1:* Based on the available information, the most appropriate destination/disposition for this patient is:

A. Ground ambulance transport to Small Local ED (no NH trauma system designation, 20 minute drive)

**B. Ground ambulance transport to Regional Trauma Center (NH Level 2 trauma center, 60 minute drive)**

**C. Air ambulance transport to Regional Trauma Center**

D. Patient self-transport to destination of choice

*Question 2:* Appropriate receiving facility pre-arrival notification includes:

**A. Trauma alert**

B. No pre-arrival notification beyond standard call, the patient does not meet alert criteria

*Confidence Scale:* My confidence in this decision using the available information is:

1. Not confident in the decision, I need more information than provided to make a confident decision

2.

3.

4.

5. Confident, the information provided was all I need to make a confident decision

*Post-Course Ultrasound Image:* Video clip of right anterior lung field with no lung sliding present

Still image of right anterior lung field M-mode with barcode sign present

Text stating “negative findings in all other eFAST windows”
