# Supplementary material for: Impact of Prehospital Ultrasound Training on Simulated Paramedic Clinical Decision-Making
Source: West J Emerg Med. 2024 Jun 28;25(5):784–92. doi: 10.5811/westjem.18439 (PMC11418876; doi:10.5811/westjem.18439)
Supplement: Supplementary file 2 [file wjem-25-784-s002.docx]

| **Topic** | **Description** | **Time Allotted** | **Student to Instructor Ratio** |
| --- | --- | --- | --- |
| Pre-Course Clinical Decision Scenarios (Written) | Pre-course scenarios were Emailed to the participants once they were registered for the course. Once completed, they were emailed the assigned prework material.  The scenarios assessed prehospital treatment, choice of receiving facility choice, transport modality and/or pre-arrival alert. Decision confidence was assessed with a Likert Scale. | As needed | N/A |
| Pre-Course Work | Carefully selected asynchronous free open access video-lecture material was sent to participants after completion of Pre-Course Clinical Decision Scenarios. Completion of course pre-work was based on the honor system and was not verified by the study team. | 3 Hours | N/A |
| Ultrasound Introduction Didactics | Content:  Physics and Knobology  Probe Maintenance  Image Acquisition | 30 Minutes | N/A  Large group |
| Cardiac Exam and Applications Didactics | Lecture Outline:  Exam Indications  Cardiac Image Acquisition  Cardiac Pathology | 40 Minutes | N/A  Large group |
| Cardiac Exam Practice | Hands on practice with portable ultrasound units on live models. | 50 Minutes | 3:1 |
| Lung Exam and Applications Didactics | Lecture Outline:  Exam Indications  Lung Image Acquisition  Lung Pathology | 40 Minutes | N/A  Large group |
| Lung Exam Practice | Hands on practice with portable ultrasound units on live models. | 50 Minutes | 3:1 |
| eFAST Exam and Applications Didactics | Lecture Outline:  Exam Indications  eFAST Image Acquisition  eFAST Pathology | 40 Minutes | N/A  Large group |
| eFAST Exam Practice | Hands on practice with portable ultrasound units on live models. | 50 Minutes | 3:1 |
| Integration Sessions (Q&A) | Multiple choice questions on cardiac, lung and eFAST image acquisition, interpretation and pathology were presented with Poll Everywhere application. Participants answered via their phone or computer and then answers were discussed as a group, including all instructors. | 1 Hour | N/A  Large group |
| Scanning Practice | Participants practiced cardiac, lung and eFAST on each other. | 20 Minutes (x3 rounds) | 6:1 |
| Practical Exam (EMS Case Scenarios) | Administration Methodology: An Instructor administered the assessment to a group of 2-3 participants at a time by running them through 6 EMS patient assessment scenarios involving cardiac, lung and eFAST exams. Each paramedic had to individually demonstrate cardiac, lung and eFAST image acquisition on a live model (free from pathology) and subsequently interpret an image with pathology viewed on a computer screen. The group of participants would decide on treatment/transport decisions based on the patient presentation in the case and the pathology image applied to each patient. | 60 Minutes  (6 Scenarios x10 minutes each) | 1:1 |
| Post Test (Written) | 24 Multiple-Choice Questions.  Question Content:  Ultrasound physics and knobology  Case-based cardiac, lung and eFAST exam indications  Ultrasound image interpretation, including normal sonographic anatomy and pathology Case-based treatment/transport decision making questions | 60 Minutes | N/A |
| Post Course Clinical Decision Scenarios (Written) | Same instrument used for “Pre-Course Clinical Decision Scenarios” with the addition of an uninterpreted ultrasound clip.  Assessed prehospital treatment, choice of receiving facility choice, transport modality and/or pre-arrival alert.  Decision confidence was assessed with a Likert Scale. | 60 Minutes | N/A |
